# Independent lung ventilation using dual ventilators for unilateral reexpansion pulmonary edema after mediastinal tumor resection: a case report

**DOI:** 10.1186/s13019-026-04247-x

**Published:** 2026-05-08

**Authors:** Yu Zhou, HongZhang Ding, Qing Yang, Teng Zheng, YuYu Gu, Feng Chen, Jinbao Li

**Affiliations:** 1 Department of Anesthesiology, Shanghai General Hospital, Shanghai Jiao Tong University School of Medicine, Shanghai, 200080 China; 2 Department of Anesthesiology, Ruijin Hospital, Shanghai Jiao Tong University School of Medicine, Shanghai, 200025 China

**Keywords:** Mediastinal tumor, Anesthesia, Reexpansion pulmonary edema, Independent lung ventilation, Case report

## Abstract

**Background:**

Reexpansion pulmonary edema following pulmonary and mediastinal surgery presents a complex clinical challenge. Independent lung ventilation (ILV), which facilitates the application of distinct positive end-expiratory pressures (PEEP) and tidal volumes to each lung, may serve as an alternative therapeutic approach for managing reexpansion pulmonary edema.

**Case presentation:**

: A 58-year-old female patient presented with a giant space-occupying lesion measuring 17.1*11.2*19.2 cm in the left lung and underwent mediastinal tumor resection under general anesthesia. Intraoperatively, following the resection of the tumor and the invaded upper lobe of the left lung, the left lung was manually reopened, resulting in the development of reexpansion pulmonary edema (RPE). To prevent exudate from the left lung from infiltrating the right lung and to avoid barotrauma to the right lung due to excessive airway pressure, a dual ventilator mechanical ventilation strategy was employed. This approach utilized a double-lumen endotracheal tube, allowing for differential ventilation modes tailored to each lung.

**Conclusion:**

The mechanical ventilation treatment involving double-lumen bronchial intubation with various ventilation modes serves as an effective ventilatory support for managing reexpansion pulmonary edema.

## Introduction

Reexpansion pulmonary edema (RPE) is a serious and potentially life-threatening complication following thoracic surgery, particularly challenging due to the absence of standardized management guidelines [1]. The pathophysiology of RPE is multifactorial and not fully elucidated, with key mechanisms including ischemia-reperfusion injury after restoration of perfusion to a hypoxic collapsed lung, increased alveolar-capillary permeability caused by mechanical stretch and inflammatory mediator release, and unfavorable hydrostatic pressure shifts during rapid lung reexpansion [2, 3]. The incidence of RPE following chest tube drainage for spontaneous pneumothorax or after thoracentesis for pleural effusion drainage is reported to be less than 1% [3, 4]. Despite its rarity, RPE is a serious complication that can lead to significant morbidity, with mortality associated with severe cases approaching 20% [5]. However, no definitive guidelines exist for the prevention or treatment of RPE [1]. Independent lung ventilation (ILV) using a double-lumen endotracheal tube is routinely used for lung isolation in thoracic procedures and has been shown effective in the management of RPE [6]. ILV permits differential application of tidal volume, positive end-expiratory pressure (PEEP), and inspiratory pressure to each lung separately, thereby shielding the unaffected lung from cross-contamination with edema fluid and reducing the risk of barotrauma. This case report describes a patient who developed unilateral RPE following mediastinal tumor resection and was successfully managed with ILV using a dual ventilator strategy.

## Case report

**Fig. 1:**
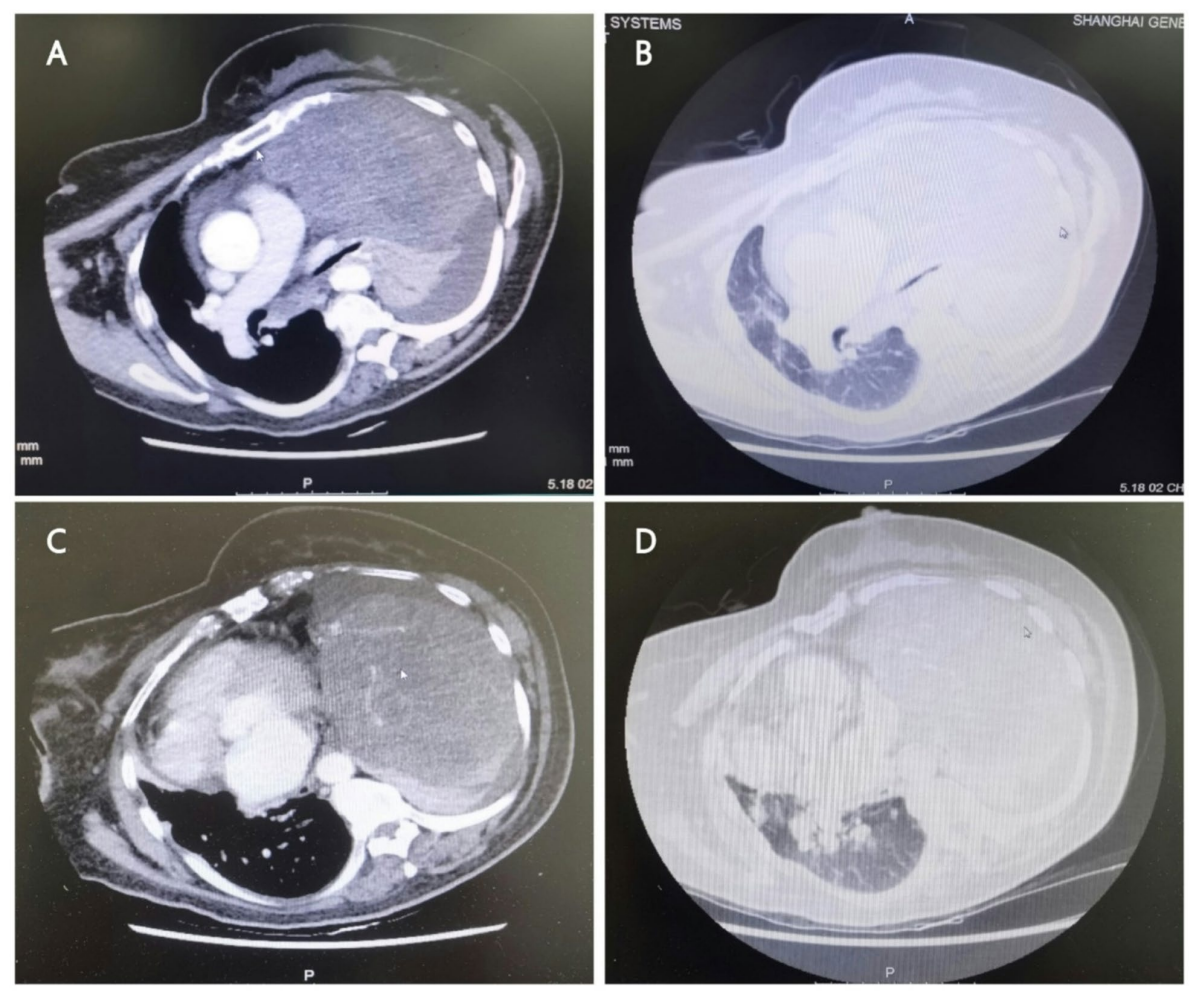
Chest CT scan preoperative. Left giant mediastinal tumor

A 58-year-old woman with hypertension and a history of subtotal thyroidectomy was found to have a 5 cm left lung tumor 8 years ago, which was not monitored. Over the past 6 months, she experienced chest tightness and dyspnea. A CT scan revealed a large left lung mass measuring 17.1*11.2*19.2 cm, identified as a solitary fibrous tumor (Fig. 1). She cannot lie flat and must maintain a forced position. Breath sounds are clear in the right lung but absent in the left, with edema in both lower limbs and limited mobility. Her SpO_2_ is 82–85%, improving to 94% with oxygen. Blood tests show hemoglobin at 107 g/L, with blood gas analysis indicating a pH of 7.44, PaCO_2_ at 54mmHg, PaO_2_ at 56mmHg, and a base excess of 12.5mmol/L. CT also shows a massive soft tissue mass, pleural effusion, left lung compression and atelectasis, enlarged lymph nodes, and thickened right pulmonary interstitium with nodules. Surgery for mediastinal tumor resection under general anesthesia is planned.

Anesthesia involved oxygen inhalation in the right lateral decubitus position, ECG monitoring, peripheral venous access, and right radial artery catheterization for arterial pressure monitoring. Autologous blood (200 ml) was reserved. Rapid induction included etomidate (0.2 mg/kg), alfentanil (20 µg/kg), and rocuronium (0.6 mg/kg), followed by left double-lumen tracheal intubation. The right internal jugular and femoral veins were catheterized, with EV1000, TEG, EEG, and body temperature monitoring. Desflurane (6%) and pure oxygen (1 L/min) were used, with a total sufentanil dose of 100 µg. The 5-hour operation resulted in fluid losses of 1100 ml pleural effusion, 200 ml pericardial effusion, 2000 ml blood, and 1500 ml urine. Transfusions included 2200 ml crystalloid, 1500 ml colloid, 6U red blood cells, 200 ml autologous blood, 600 ml fresh frozen plasma, 40 g albumin, 4U cryoprecipitate, 1U platelets, 1 g fibrinogen, and 600U prothrombin complex.

**Fig. 2:**
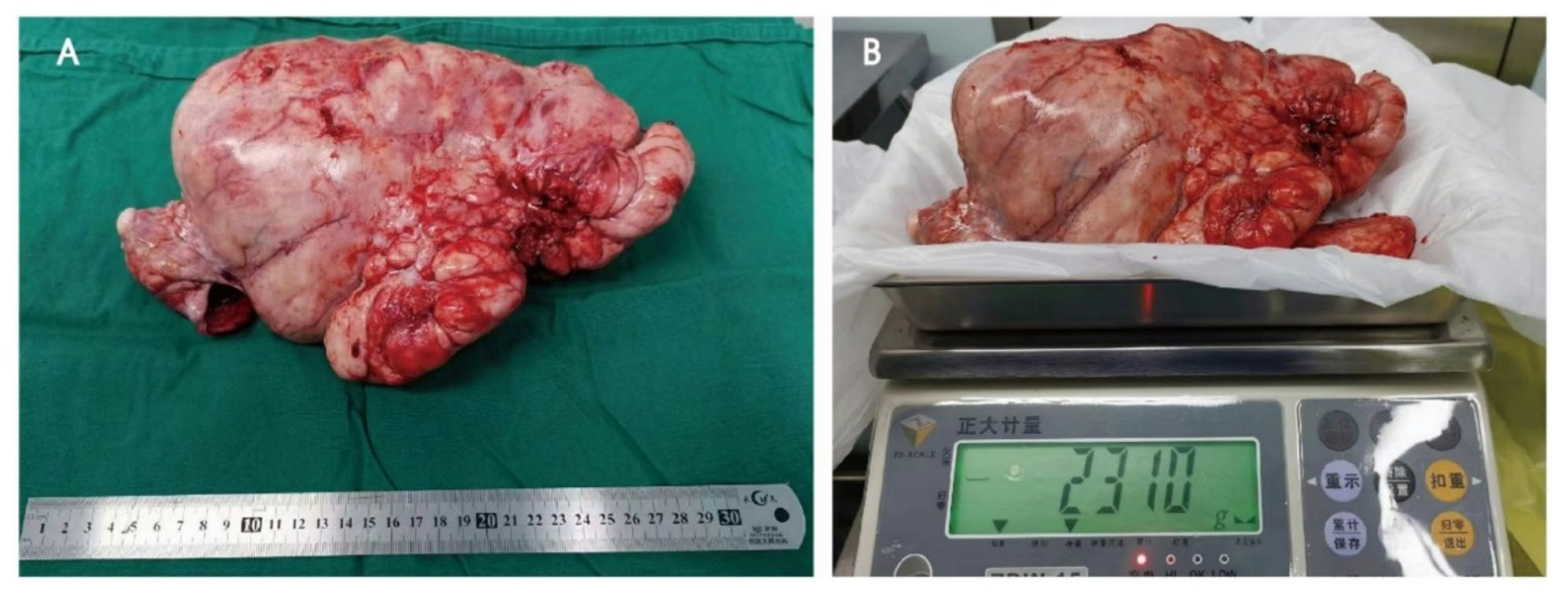
Tumor specimen removed during surgery

A transverse sternal Clamshell incision was performed to access a 30 cm mass in the anterior upper mediastinum, which had invaded the bilateral mediastinal pleura, the left lung’s upper lobe, and parts of the right lung’s upper, middle, and lower lobes. The tumor’s upper and lower poles were separated, and the mass, along with the affected lung sections, was excised (Fig. 2). Bleeding was controlled, chest tubes were placed bilaterally, and the chest was closed in layers.

**Fig. 3:**
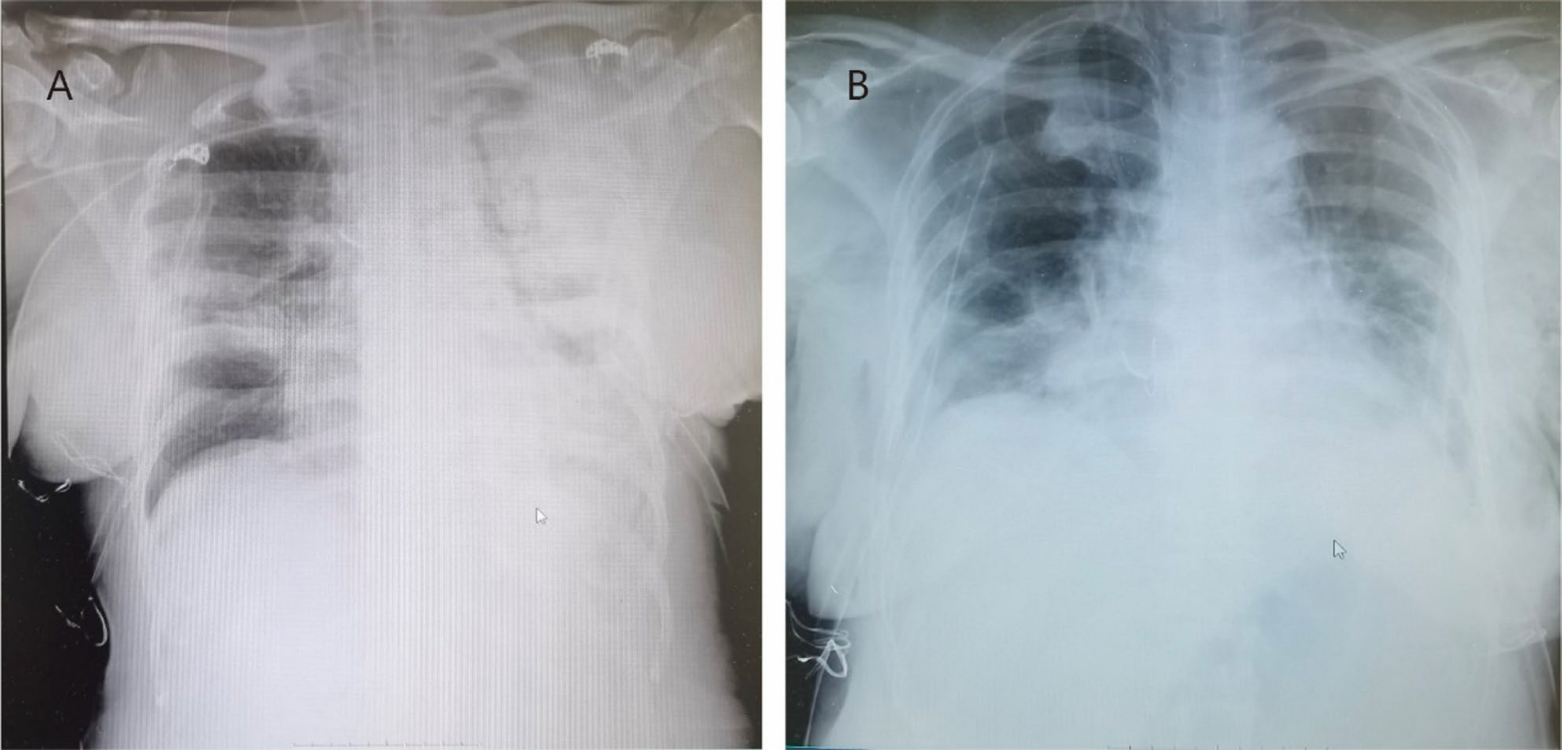
Chest radiograph obtained on the first day(A) and fifth day(B) after surgery

During the operation, after removing the tumor and the upper lobe of the invaded left lung, the left lung was reopened and found to be severely congested with a liver-like texture. A large amount of pink liquid exuded from the left double lumen tube, and the airway pressure was extremely high at 50–60 cmH_2_O, making it difficult to maintain oxygen saturation. To complete the operation, intermittent ventilation was used on the right lung after partial resection of its lobes. To prevent left lung exudate from affecting the right lung and avoid air pressure injury, a dual ventilator mechanical ventilation strategy was implemented, using different ventilation modes for each lung. For treating the left lung’s reexpansive pulmonary edema, the settings were: tidal volume at 200 ml, PEEP at 12 cmH_2_O, and a respiratory rate of 14 breaths per minute. Propofol and sufentanil were administered for sedation, and pancuronium at 4 mg/hour was used for muscle relaxation in the ICU. After 18 h of mechanical ventilation, a chest X-ray, lung auscultation, bronchoscopy for left lung edema, and blood gas analysis were performed (Fig. 3). Once the left lung edema improved, the double lumen endotracheal tube was replaced with a single lumen tube, and breathing support continued. Respiratory function exercises began after 42 h, and the tracheal tube was removed after 66 h, switching to nasal oxygen. The patient returned to the general ward on the fourth day post-operation and was discharged successfully on the 11th day.

## Discussion

### Preoperative anesthesia planning

Perioperative anesthetic management of patients with mediastinal masses remains challenging due to potential airway compression, circulatory instability, and respiratory failure. Several strategies have been proposed to improve safety in such patients [7]. After multidisciplinary evaluation, the following key decisions were made for this patient: (1) The patient presented with a large, long-standing mediastinal mass causing severe compressive symptoms, diagnosed as solitary fibrous tumor, with no indication for preoperative chemoradiotherapy. (2) Preoperative chest CT demonstrated severe atelectasis of the left lung due to external compression rather than direct tumor invasion, supporting an attempt at lung preservation during tumor resection. (3) Incidental small nodules in the right lung were planned for simultaneous resection.

The patient was unable to lie supine, with baseline SpO₂ 82–85% (increased to 94% with oxygen supplementation). Arterial blood gas analysis showed hypercapnia (PaCO₂ 54 mmHg) and hypoxemia (PaO₂ 56 mmHg), indicating severe chronic hypoventilation. Although awake intubation with a Uniblocker and spontaneous ventilation may be safer in patients with large mediastinal masses [8], recent evidence suggests that airway collapse is not inevitable during induction [9]. In this case, CT showed no critical tracheobronchial narrowing. Given the patient’s inability to lie flat, a double-lumen tube was inserted in the lateral position after preoxygenation.

Facial edema suggested possible superior vena cava syndrome, which may deteriorate during tumor dissection. Right internal jugular vein catheterization was performed for continuous central venous pressure monitoring, and right femoral vein cannulation was prepared for rapid transfusion. Right radial artery cannulation was established before anesthesia for real-time blood pressure monitoring and to avoid vascular injury during tumor dissection. Continuous EEG monitoring was used to detect potential cerebral complications related to elevated intracranial venous pressure. Severe cardiac compression and displacement, combined with left lung atelectasis, increased the risk of cardiac arrest. Therefore, left femoral vessels were prepared for potential emergency cannulation, and the cardiac surgery team was on standby for extracorporeal circulation support if needed.

### Interpretation of preoperative blood gas and perioperative risk assessment

Preoperative arterial blood gas analysis showed hypercapnia (PaCO₂ 54 mmHg), hypoxemia (PaO₂ 56 mmHg on room air), elevated base excess (12.5 mmol/L), and normal pH (7.44). This pattern indicated chronic respiratory acidosis with metabolic compensation resulting from long-term hypoventilation caused by mass compression. Such chronic respiratory impairment significantly increases perioperative risk, including reduced pulmonary reserve, higher susceptibility to anesthetic-induced respiratory depression, and risk of postoperative respiratory failure. Based on this risk assessment, anesthetics were carefully titrated, postoperative mechanical ventilation was planned, and serial blood gas analyses were used to guide ventilator weaning. This strategy contributed to successful extubation on postoperative day 3.

### Intraoperative challenges and management

After careful preoperative preparation, anesthesia induction and supine positioning were completed uneventfully. A transverse sternal Clamshell incision was used for tumor resection. Two major intraoperative challenges were encountered: (1) The firm fibrous tumor required blunt dissection, which repeatedly compressed the heart and great vessels, leading to transient hemodynamic instability. This was managed by deepening anesthesia, enhancing analgesia, and temporarily pausing dissection to relieve compression, allowing gradual hemodynamic recovery. (2) After removal of the mass, reinflation of the chronically collapsed left lung was immediately followed by profuse pink frothy fluid from the left bronchus, along with extremely high airway pressure (50–60 cmH₂O) and refractory hypoxemia. During right pulmonary nodule resection, intermittent single-lung ventilation of the right lung was applied. Postoperatively, bilateral ventilation via a double-lumen tube revealed persistent high airway pressure and pink frothy secretion from the left lung, confirming severe reexpansion pulmonary edema (RPE). Conversion to a single-lumen tube or tracheotomy was considered high risk for barotrauma and cross-contamination. Therefore, independent lung ventilation (ILV) using dual ventilators was adopted, as supported by previous reports [10, 11].

### Technical details of the dual ventilator strategy

To ensure reproducibility of our ILV strategy, the following technical specifications are provided. Two separate ICU ventilators (Draeger Evita XL, Germany) were used. For treating the left lung’s reexpansion pulmonary edema, the edematous lung was ventilated using pressure-controlled ventilation (PCV) mode to limit peak airway pressure, with inspiratory pressure set at 25 cmH₂O, PEEP 12 cmH₂O, tidal volume 200 ml (resulting), and a respiratory rate of 14 breaths per minute. The healthy right lung was ventilated using volume-controlled ventilation (VCV) mode to ensure consistent tidal volume delivery, with tidal volume 400 ml, PEEP 5 cmH₂O, and the same respiratory rate of 14 breaths per minute.

The two ventilators were not mechanically synchronized; instead, continuous deep sedation (propofol 2–4 mg/kg/h) and neuromuscular blockade (pancuronium 4 mg/h) were administered to eliminate spontaneous breathing and prevent mediastinal swing. The inspiratory/expiratory ratio was set identically at 1:2 on both ventilators to promote synchronous chest wall movement. Correct positioning of the left-sided double-lumen tube (37 Fr) was confirmed by fiberoptic bronchoscopy before initiating ILV, and cuff pressures were maintained at 25–30 cmH₂O to prevent cross-ventilation between the two lungs. Each lumen was connected exclusively to its designated ventilator with no shared circuits, and continuous capnography was monitored on each ventilator to detect any air leakage or cross-ventilation.

### Establishment of RPE diagnosis and exclusion of ARDS and TRALI

The diagnosis of RPE was established according to typical clinical, radiologic, and gas exchange criteria: (1) Trigger and timing: Acute pulmonary edema developed immediately after reinflation of the long-term atelectatic left lung, the classic temporal feature of RPE. (2) Clinical findings: Direct visualization showed severe congestion, liver-like consistency, and copious pink frothy exudate from the left bronchus. (3) Radiographic features: Chest imaging demonstrated unilateral infiltrates limited to the left lung, without bilateral diffuse opacities. (4) Oxygenation: Severe hypoxemia with reduced PaO₂/FiO₂ ratio was present, which improved rapidly after ILV with high PEEP.

ARDS was excluded based on the Berlin definition: infiltrates were unilateral rather than bilateral; edema was directly caused by lung reexpansion, not systemic inflammatory injury; and no other ARDS-predisposing conditions were present.

TRALI was excluded because acute hypoxemia occurred immediately after lung reexpansion, with no temporal relationship to blood transfusion; edema was strictly unilateral; and there were no typical features of TRALI such as bilateral infiltrates or acute respiratory failure temporally associated with transfusion.

Although substantial fluid resuscitation and blood transfusion were given intraoperatively due to extensive bleeding and effusion drainage, hemodynamic monitoring (central venous pressure, EV1000) showed no evidence of heart failure or elevated pulmonary capillary wedge pressure. Moreover, edema developed before most fluid and transfusion therapy was completed, and the patient responded rapidly to ILV with high PEEP rather than diuresis or inotropic support.

Therefore, the primary diagnosis was reexpansion pulmonary edema, and ARDS, TRALI, cardiogenic pulmonary edema, and fluid overload were reasonably excluded as the main cause.

### Risk factors for RPE in this patient

This patient presented multiple well-documented risk factors for RPE. First, the left lung sustained chronic, massive atelectasis for several years due to compression from a giant mediastinal mass, which is the most established risk factor for RPE. Second, acute, rapid manual reexpansion immediately after tumor removal further increased pulmonary vascular permeability and triggered alveolar fluid exudation. Third, prolonged hypoxemia and hypercapnia indicated chronic respiratory impairment and reduced pulmonary reserve. Fourth, repeated intraoperative circulatory fluctuations due to tumor compression may have further contributed to lung injury. The combination of these factors directly led to the development of severe RPE in this case.

### Rationale for ILV versus conventional lung-protective ventilation

Conventional lung-protective ventilation using a single lumen tube was insufficient for this patient with severe unilateral RPE. Conventional ventilation cannot provide separate ventilatory parameters for each lung; high pressure required to recruit the edematous lung would cause barotrauma in the healthy right lung. In addition, pink frothy exudate from the injured lung carries a high risk of contaminating the contralateral lung and worsening oxygenation.

In contrast, independent lung ventilation (ILV) allows differential management: the injured left lung was supported with low tidal volume and high PEEP to reduce edema, while the healthy right lung was ventilated with standard protective parameters to maintain gas exchange and avoid injury. ILV also prevents cross-contamination and ensures hemodynamic stability. Therefore, ILV was the only rational and effective strategy for this life-threatening condition.

### Potential complications of ILV and preventive strategies

ILV is associated with several potential complications, including mediastinal swing from asynchronous ventilation, barotrauma, double-lumen tube malposition, airway injury, and ventilator-associated pneumonia.

In this case, we implemented strict preventive measures: continuous sedation and muscle relaxation were used to eliminate asynchronous breathing and stabilize the mediastinum (compensating for the lack of mechanical synchronization); airway pressures were strictly limited by using pressure-controlled mode on the injured lung; fiberoptic bronchoscopy was used to confirm correct double-lumen tube position and to prevent cross-ventilation; and intensive airway care was provided to prevent infection. No ILV-related complications occurred during treatment.

### Comparison with previously published cases

Previous studies and case reports have demonstrated that ILV is effective for refractory RPE after thoracic surgery, thoracentesis, or pneumothorax drainage. Existing evidence confirms that ILV enables differential ventilation, protects the healthy lung from barotrauma, avoids cross-contamination, and rapidly improves oxygenation. Consistent with published literature, our case also showed that ILV rapidly improved oxygenation and resolved severe RPE without tracheotomy.

Compared with previous reports, our case had several unique features: RPE occurred following resection of a giant mediastinal tumor with long-standing massive atelectasis; the patient had severe preoperative respiratory dysfunction and hypercapnia and could not lie supine, representing a high-risk population rarely described; and ILV was initiated intraoperatively and continued postoperatively as an emergency rescue therapy rather than starting in the ICU postoperatively. Our PEEP strategy (12 cmH₂O for the injured lung) was consistent with levels used in similar cases (8–15 cmH₂O). This case not only validates the existing evidence but also expands the application scope of ILV to RPE complicating giant mediastinal tumor resection, further supporting that ILV is a reliable and minimally invasive salvage approach for severe RPE.

### Postoperative complication management

In ICU, ILV was applied via a double-lumen tube with separate ventilator settings for each lung. For the edematous left lung, low tidal volume (200 ml), relatively high PEEP (12 cmH₂O), and respiratory rate 14 breaths/min were used to reduce extravasation. For the right lung, tidal volume 400 ml, PEEP 5 cmH₂O, and the same respiratory rate were applied to maintain oxygenation while avoiding barotrauma. Unlike cardiogenic pulmonary edema, RPE is not associated with elevated pulmonary capillary wedge pressure (< 18 mmHg) [12], justifying the use of moderate-to-high PEEP in this patient. Continuous sedation and muscle relaxation were used to prevent mediastinal swing and asynchronous breathing.

Weaning from ILV requires careful assessment [6]. After 18 h of mechanical ventilation, chest radiography, auscultation, and fiberoptic bronchoscopy showed significant resolution of left-sided pulmonary edema. The double-lumen tube was then replaced with a single-lumen tube. Respiratory rehabilitation was initiated at 42 h. After 66 h of mechanical ventilation, the patient was successfully extubated and switched to nasal oxygen. The patient was transferred to the general ward on postoperative day 4 and discharged on day 11.

## Conclusion

In patients with reexpansion pulmonary edema following resection of giant mediastinal tumors, independent lung ventilation using a double-lumen endotracheal tube with individualized ventilator parameters provides effective respiratory support. This strategy helps avoid tracheotomy and prolonged ventilation, representing a minimally invasive and effective salvage approach for severe RPE.

## Data Availability

No datasets were generated or analysed during the current study.
